# Epitranscriptomic influences on development and disease

**DOI:** 10.1186/s13059-017-1336-6

**Published:** 2017-10-23

**Authors:** Phillip J. Hsu, Hailing Shi, Chuan He

**Affiliations:** 1 Department of Chemistry and Institute for Biophysical Dynamics, Howard Hughes Medical Institute, The University of Chicago, Chicago, IL 60637 USA; 2 Medical Scientist Training Program and Committee on Immunology, The University of Chicago, Chicago, IL 60637 USA; 3 Department of Biochemistry and Molecular Biology, The University of Chicago, Chicago, IL 60637 USA

## Abstract

RNA contains over 150 types of chemical modifications. Although many of these chemical modifications were discovered several decades ago, their functions were not immediately apparent. Discoveries of RNA demethylases, along with advances in mass spectrometry and high-throughput sequencing techniques, have caused research into RNA modifications to progress at an accelerated rate. Post-transcriptional RNA modifications make up an epitranscriptome that extensively regulates gene expression and biological processes. Here, we present an overview of recent advances in the field that are shaping our understanding of chemical modifications, their impact on development and disease, and the dynamic mechanisms through which they regulate gene expression.

## Introduction

Over 150 unique chemical modifications of RNA have been found in different organisms. The first of these modifications was discovered in 1951, when ion-exchange analysis of RNA revealed an abundant unknown modification later identified as pseudouridine (Ψ) [1–4]. Discoveries of other abundant modifications using radioactive labeling followed: 2′-*O*-methylation (2′OMe) and *N* ^1^-methyladenosine (m^1^A) were discovered in tRNA and ribosomal RNA (rRNA); and 2′OMe, *N* ^6^-methyladenosine (m^6^A) and 5-methylcytidine (m^5^C) were found in mRNA and viral RNA [5–8]. As the modifications were systematically characterized and catalogued, hints to their functions emerged. m^6^A, the most abundant internal modification of eukaryotic mRNA, was shown in early studies to facilitate the processing of pre-mRNA and the transport of mRNA [9, 10].

We proposed previously that post-transcriptional RNA modifications could be reversible and may significantly impact the regulation of gene expression [11]. This hypothesis was confirmed with the discovery of fat-mass and obesity-associated protein (FTO), the first enzyme known to demethylate m^6^A on RNA, soon followed by that of alkB homologue 5 (ALKBH5), a second m^6^A demethylase [12, 13]. In 2012, m^6^A-specific antibodies were used to profile m^6^A sites through immunoprecipitation followed by high-throughput sequencing. Thousands of m^6^A sites were identified in human and mouse cell lines, with enrichment around the stop codon and 3′ UTR [14, 15]. These advances sparked extensive research on RNA post-transcriptional modifications in this new era of epitranscriptomics. In this review, we summarize the most recent advances in the field, focusing on functional investigations.

## m^6^A writers and readers lead the way

**Fig. 1:**
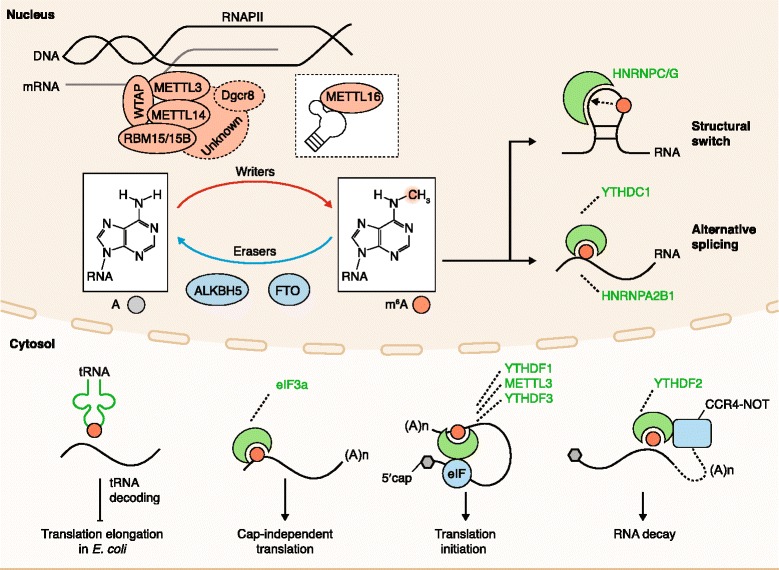
The m^6^A machinery. The writers, readers, erasers, and cellular components of eukaryotes that interact with m^6^A and the RNA that contains it. *A* adenosine, *ALKBH5* AlkB homologue 5, *eIF3* eukaryotic initiation factor 3, *FTO* fat-mass and obesity-associated protein, *HNRNPC* heterogeneous nuclear ribonucleoprotein C; *m* ^*6*^ *A N* ^6^-methyladenosine, *METTL3* methyltransferase-like 3, *RNAPII* RNA polymerase II, *YTHDC1* YTH domain containing 1, *YTHDF1* YTH domain family 1

m^6^A is installed by a methyltransferase complex that includes the *S-*adenosyl methionine (SAM) binding protein methyltransferase-like 3 (METTL3), first identified over two decades ago [16, 17] (Fig. 1). Recent experiments have established that METTL3 and METTL14 are essential components of a writer complex, in which METTL3 is catalytically active while METTL14 has critical structural functions [18, 19]. Functional roles of m^6^A were discovered through experiments in which METTL3 was inactivated; these studies showed that loss of m^6^A compromises circadian rhythm, embryonic stem cell fate transition, and naïve pluripotency [20–22]. A new m^6^A methyltransferase, METTL16, has been shown to regulate the splicing of the human SAM synthetase MAT2A, promoting its expression through enhanced splicing of a retained intron in SAM-depleted conditions, and thus acting as a regulation loop [23]. METTL16 was also shown to be the m^6^A methyltransferase of the U6 small nuclear RNA.

Importantly, m^6^A regulates gene expression through various m^6^A-recognition proteins. YTH domain containing 1 (YTHDC1), an m^6^A ‘reader’, acts in the nucleus to influence mRNA splicing [24], whereas heterogeneous nuclear ribonucleoprotein C (HNRNPC) and HNRNPG bind to RNAs whose structures have been altered by m^6^A to promote mRNA processing and alternative splicing [25, 26]. In the cytosol, the m^6^A readers YTH domain family 1 (YTHDF1) and YTHDF3 affect the translation of their targets through ribosome loading in HeLa cells [27–29], and YTHDF2 facilitates mRNA degradation by recruiting the CCR4-NOT deadenylase complex [30, 31]. The m^6^A reader YTHDC2 also functions in the cytosol, affecting the translation efficiency and mRNA abundance of its targets [32]. As research elucidates the functions of m^6^A readers, it is becoming evident that their roles may be complex. m^6^A in the 5′ UTR could facilitate cap-independent translation initiation through a process involving eIF3 [33, 34]. The exact ‘reading’ mechanism of this process is still unclear. Under heat shock, YTHDF2 shields 5′ UTR m^6^A from FTO, allowing selective mRNA translation. It will be important to determine the functional roles of readers under different biological conditions.

## Effects of m^6^A at the molecular level

m^6^A appears to influence almost every stage of mRNA metabolism. Three recent studies demonstrated interactions with the translation, transcription, and microprocessor machineries (Fig. 1). In an *Escherichia coli* translation system, the presence of m^6^A on mRNA interferes with tRNA accommodation and translation elongation [35]. Although m^6^A does not interfere with the structure of the codon–anticodon interaction, minor steric constraints destabilize base-pairing. The magnitude of the resulting delay is affected by the position of the m^6^A, implying that m^6^A may be an important regulator of tRNA decoding. m^6^A was also shown to be correlated with decreased translation efficiency in a study using MCF7 cells [36]. In this experiment, an inducible reporter system was used to demonstrate that transcripts with slower rates of transcription received greater deposition of m^6^A, and that m^6^A deposition occurs co-transcriptionally. This work also showed that METTL3 interacts with RNA polymerase II under conditions of slower transcription, and that methylated transcripts had decreased efficiency of translation. As m^6^A has been shown to promote translation in other studies [27, 33, 34], the role of m^6^A in affecting translation could be transcript- and position-dependent. Although the m^6^A itself could reduce translation efficiency, as shown in the in vitro experiment [35], the YTH domain proteins could promote translation in response to stimuli or signaling. A recent study showed that METTL3 binds to RNA co-transcriptionally, and that this interaction is necessary for the microprocessor components Dgcr8 and Drosha to associate physically with chromatin to mediate gene silencing [37]. METTL3 and Dgcr8 relocalize to heat-shock genes under hyperthermia and work in concert to promote the degradation of their targets, allowing timely clearance of heat-shock responsive transcripts after heat-shock has ended. These studies reveal important roles for m^6^A in enhancing the dynamic control of gene expression, a function that is especially important under changing cell conditions.

## Influences of m^6^A on development and differentiation

We recently proposed that m^6^A shapes the transcriptome in a manner that facilitates cell differentiation [38]. Such a role could be critical during development, as is suggested by several recent studies. m^6^A is necessary for sex determination in *Drosophila* [39, 40]. Depletion of the *Drosophila* METTL3 homologue Ime4 leads to the absence of m^6^A on the sex determination factor *Sex lethal* (*Sxl*). Without m^6^A, the YTHDC1 homologue YT521-B is unable to properly splice *Sxl*, leading to failure of X inactivation and thus improper sex determination. Moreover, depletion of Ime4 affects neuronal function, causing shortened lifespan and irregularities in flight, locomotion, and grooming. m^6^A has also been shown to regulate the clearance of maternal mRNA during the maternal-to-zygotic transition in zebrafish [41]. Zebrafish embryos that lack the m^6^A reader Ythdf2 become developmentally delayed because of impaired decay of m^6^A-modified maternal RNAs. Because these maternal RNAs are not properly decayed, activation of the zygotic genome is also impaired.

Previous studies have demonstrated roles for m^6^A in the differentiation of mouse and human embryonic stem cells [21, 22, 42]. More recently, effects of m^6^A on differentiation have been shown in mice. Two separate studies showed that the meiosis-specific protein MEIOC, which is necessary for proper meiotic prophase I during spermatogenesis, interacts with the m^6^A reader YTHDC2 [43, 44]. Mice that lack *Meioc* are infertile, lacking germ cells that have reached the pachytene phase of meiotic prophase I. Notably, mice lacking *Ythdc2* or *Mettl3* display similar phenotypes, demonstrating infertility and defects in germ cells, which reach a terminal zygotene-like stage and undergo apoptosis [32, 45]. m^6^A also affects somatic cell differentiation in mice. Knockout of *Mettl3* in mouse T cells caused failure of naïve T cells to proliferate and differentiate; in a lymphopaenic adoptive transfer model, most naïve *Mettl3*-deficient T cells remained naïve, and no signs of colitis were present [46]. The lack of *Mettl3* caused upregulation of SOCS family proteins, which inhibited the IL-7-mediated STAT5 activation necessary for T cell expansion. Two studies of FTO have also demonstrated roles for m^6^A in somatic cell differentiation. FTO expression was shown to increase during myoblast differentiation, and its depletion inhibited differentiation in both mouse primary myoblasts and mouse skeletal muscle [47]. The demethylase activity of FTO is required: a point mutation of FTO that removes demethylase activity impairs myoblast differentiation. FTO is also dynamically expressed during postnatal neurodevelopment, and its loss impedes the proliferation and differentiation of adult neural stem cells [48].

## Involvement of m^6^A in human cancer

As discussed in the previous section, m^6^A is a critical factor in cell differentiation. Considering that cancer is driven by the misregulation of cell growth and differentiation, it follows that cancer cells may hijack aberrant methylation to enhance their survival and progression. Several studies have demonstrated roles for demethylation or lack of methylation in promoting cancer progression. In *MLL*-rearranged acute myeloid leukemia (AML), FTO is highly expressed, promotes oncogene-mediated cell transformation and leukemogenesis, and inhibits all-*trans*-retinoic acid (ATRA)-induced AML cell differentiation [49]. At the molecular level in AML, FTO causes both a decrease in m^6^A methylation and a decrease in the transcript expression of these hypo-methylated genes. *ASB2* and *RARA* are functionally important targets of FTO in *MLL*-rearranged AML; their forced expression rescues ATRA-induced differentiation. The oncogenic role of FTO is not limited to AML; another study showed that inhibition of FTO in glioblastoma stem cells (GSCs) suppresses cell growth, self-renewal, and tumorigenesis [50]. This study demonstrated that other components of m^6^A machinery also impact glioblastoma. Knockdown of METTL3 or METTL14 affects the mRNA expression of genes that are crucial to GSC function, and enhances GSC growth, proliferation, and tumorigenesis. In agreement with these findings that lack of methylation tends to promote cancer progression, Zhang et al. [51] showed that ALKBH5 is highly expressed in GSCs, and that its knockdown suppresses their proliferation. The protein abundance of the ALKBH5 target *FOXM1* is greatly increased in GSCs as a result of the demethylation activity of ALKBH5; removal of m^6^A at the 3′ end of *FOXM1* pre-mRNA promotes *FOXM1* interaction with HuR, which enhances FOXM1 protein expression. A long non-coding RNA (lncRNA) antisense to *FOXM1* facilitates the interaction between ALKBH5 and *FOXM1*, and depletion of either ALKBH5 or its antisense lncRNA inhibits GSC tumorigenesis. ALKBH5 also promotes a breast cancer phenotype; under hypoxic conditions, ALKBH5 expression increases, thus decreasing levels of m^6^A and upregulating expression of the pluripotency factor NANOG [52].

Together, the studies mentioned above suggest that a decrease in RNA m^6^A methylation tends to facilitate cancer progression, and that RNA methylation could affect cell growth and proliferation. Other studies, however, indicate that the role of m^6^A in different cancers may be more complex. In hepatocellular carcinoma (HCC), METTL14 downregulation is associated with tumor metastasis, but METTL3 enhances the invasive ability of HCC cells [53]. Several other studies also point to an oncogenic role for the methyltransferase complex. METTL3 plays an oncogenic role in cancer cells, promoting the translation of cancer genes through interactions with the translation initiation machinery [54]. Interestingly, METTL3 promotes translation independent of its methyltransferase activity or of any interaction with the m^6^A reader YTHDF1. WTAP, a component of the m^6^A methyltransferase complex, also promotes leukemogenesis, and its levels are increased in primary AML samples [55]. RBM15, another methyltransferase complex component, is altered in acute megakaryoblastic leukemia, undergoing translocation to fuse with *MKL1* [56].

Considering the complex findings, it is likely that different types of cancers can be derived from unique imbalances or misregulation of mRNA methylation. In AML, increased WTAP and RBM15 expression (or writer proteins themselves) could block differentiation, leading to leukemia, whereas increased eraser expression could cause leukemia via separate pathways. The intricate network of interactions is reminiscent of studies of DNA methylation; just as misregulation of DNMT and TET proteins are both associated with cancer [57–60], misregulation of the m^6^A machinery can lead to cancer through unique mechanisms. Interestingly, the oncometabolite D-2-hydroxyglutarate (D2-HG), which could act as a nonspecific inhibitor of the iron- and αKG-dependent dioxygenases FTO and ALKBH5, accumulates in about 20% of AMLs [61], and may thus contribute to the outcome of these cancers by inhibiting RNA demethylation. Further investigation is necessary to uncover mechanisms by which aberrant methylation affects the proliferation of various cancers.

## Other modifications on mRNA

**Fig. 2:**
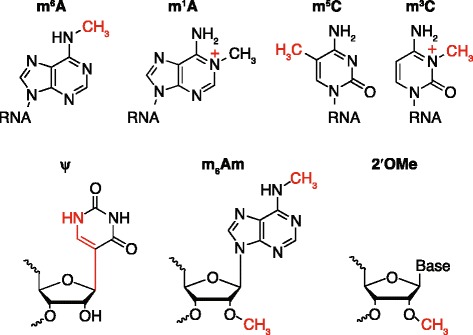
Chemical modifications of RNA in eukaryotes. Chemical structures characterized as modifications of eukaryotic RNA

Recent advances in high-throughput sequencing and mass spectrometry have revitalized research on post-transcriptional modifications, elucidating functions of both known and newly discovered modifications on mRNA (Fig. 2).

Methylation of the *N* ^1^ position of adenosine (m^1^A) was recently discovered on mRNA; this modification was found to occur on RNA at levels around 10–30% of that of m^6^A, depending on the cell line or tissue [62, 63]. m^1^A occurs in more structured regions and is enriched near translation initiation sites. The level of m^1^A responds dynamically to nutrient starvation and heat shock, and the 5′ UTR peaks correlate with translation upregulation. As it is positively charged, the m^1^A modification may markedly alter RNA structure as well as RNA interactions with proteins or other RNAs. Zhou et al. [64] demonstrated that m^1^A causes A-U Hoogsteen base pairs in RNA to be strongly disfavored, and that RNA that contains m^1^A tends to adopt an unpaired anti conformation. m^1^A was also shown to affect translation; its presence at the first or second codon position, but not at the third codon, blocks translation in both *Escherichia coli* and wheat germ extract systems [65]. In addition, m^1^A is present in early coding regions of transcripts without 5′ UTR introns, which are associated with low translation efficiency and which facilitate noncanonical binding by the exon junction complex [66]. These studies point to a main role of m^1^A in translation and RNA–RNA interactions. The exact functional roles of 5′ UTR m^1^A sites require further studies, and there are also other m^1^A sites in mRNA that could play distinct roles. Methods to map low abundance m^1^A sites in mRNA will be crucial to understanding their biological roles [67].

Adenosines at the second base of mRNAs can also undergo both 2′-*O*-methylation and m^6^A methylation to become m^6^A_m_, a modification with an unidentified methyltransferase [68, 69]. m^6^A_m_ was recently profiled at single-nucleotide resolution by crosslinking RNA to m^6^A antibodies and then identifying mutations or truncations in reverse transcription by high-throughput sequencing [70]. It undergoes preferential demethylation by FTO. The study by Mauer et al. [70] revealed negligible effects of FTO on internal mRNA m^6^A in vitro and inside cells. However, this is not consistent with the findings of many previous biochemical and cell-based studies [12, 34, 49, 71, 72]; clear sequential m^6^A demethylation by FTO has been demonstrated biochemically [71]. FTO works on both m^6^A and m^6^A_m_, with greater demethylase activity toward m^6^A modifications that are located internally on mRNA when ultra-performance liquid chromatography-tandem mass spectrometry (UHPLC-MS/MS) is used to quantify modification changes in a range of different cell lines. Because FTO can work on multiple substrates, including m6Am, and m6Am methylation occurs on only a fraction of all mRNA [73], it will be critical to determine the functional relevance of m6Am demethylation as has been done with internal m6A demethylation [34, 49, 72]. The methyltransferase will need to be identified and the phenotypes of knockout mice and cell lines will need to be examined carefully.

Cytosine methylations are also prevalent in RNA. m^5^C was first identified on RNA more than 40 years ago, and is present in all three domains of life [74]. It has been sequenced on mRNA using bisulfite sequencing, and was found to be highly prevalent in both coding and non-coding RNA [75, 76]. Bisulfite sequencing of m^5^C on mRNA may, however, produce false positives due to incomplete deamination of unmodified cytidines. Although several biological functions of m^5^C have been discovered on tRNA (as discussed in the following section), the biological functions of m^5^C in mRNA have remained largely elusive. Recently, however, a function of m^5^C on mRNA was recently discovered by Yang et al. [77]: m^5^C promotes nuclear export because it is specifically recognized by the mRNA export adaptor ALYREF. Notably, the study by Yang et al. [77] found enrichment of m^5^C sites located 100 nucleotides after translation initiation sites, which were not observed by previous studies. Further studies on the enzymes that interact with m^5^C may lead to the discovery of additional roles for m^5^C in mRNA.

3-Methylcytosine was recently identified as a modification in mRNA, present at a rate of around 0.004% of cytosines in human cell cultures [78]. It is installed by METTL8, and its function and localization have yet to be identified.

Pseudouridine, which is generated by isomerization of uridine, is the most abundant RNA modification in total RNA [3]. It was recently identified on mRNA and mapped by several groups using similar techniques (PseudoU-seq, Ψ-seq, PSI-seq, and CeU-seq), which use the water-soluble diimide CMCT (1-cyclohexyl-3-(2-morpholinoethyl)-carbodiimide metho-p-toluenesulfonate) to generate strong reverse transcriptase stops at ψ sites [79–82]. PseudoU-seq and Ψ-seq identified > 200 and > 300 sites, respectively, on human and yeast mRNAs, and Ψ/U in mRNA has been quantified at around 0.2–0.7% in mammalian cell lines. Direct evidence of biological functions of Ψ on mRNA has yet to be identified, but several findings point to potential biological roles. Ψ affects the secondary structure of RNA and alters stop codon read through [83, 84]. Depletion of the pseudouridine synthase PUS7 decreases the abundance of mRNAs containing Ψ, suggesting that Ψ may also affect transcript stability [80]. Moreover, pseudouridinylation on transcripts is affected by stresses such as heat shock and nutrient deprivation, suggesting that Ψ may be a response to various stresses [79, 80, 82].

## Modifications on transfer RNAs and other RNAs

tRNAs contain more modifications than any other RNA species, with each tRNA containing, on average, 14 modifications [74]. Recent studies have identified tRNA demethylases and methyltransferases, as well as the functions of their modifications.

Liu et al. [85] recently identified a tRNA demethylase for the first time; ALKBH1 demethylates m^1^A58 in tRNA^iMet^ and several other tRNA species. m^1^A58 increases tRNA^iMet^ stability, and its demethylation by ALKBH1 decreases the rate of protein synthesis. A related demethylase, ALKBH3, removes m^6^A from tRNA and increases translation efficiency in vitro, though its cellular targets and functions have yet to be identified [86].

m^5^C on tRNA can also influence translation, particularly affecting stress responses. Deletion of the tRNA m^5^C methyltransferase NSUN2 reduces tRNA m^5^C levels and promotes cleavage of unmethylated tRNAs into fragments, which decrease protein translation rates and induce stress response pathways [87]. Lack of *Nsun2* in mice leads to an increase in undifferentiated tumor stem cells due to decreased global translation, which increases the self-renewal potential of the tumor-initiating cells [88]. Interestingly, lack of *Nsun2* also prevents cells from activating survival pathways when treated with cytotoxic agents, suggesting that the combination of m^5^C inhibitors and chemotherapeutic agents may effectively treat certain cancers.

m^5^C also plays an important role in the translation of the mitochondrial tRNA for methionine (mt-tRNA^Met^). m^5^C is deposited onto cytosine 34 of mt-tRNA^Met^ by the methyltransferase NSUN3 [89–91]. Lack of NSUN3 leads to deficiencies such as reduced mitochondrial protein synthesis, reduced oxygen consumption, and defects in energy metabolism. Mutation of NSUN3 is also associated with several diseases, including maternally inherited hypertension and combined mitochondrial respiratory chain complex deficiency. Mechanistically, m^5^C is oxidized by ALKBH1/ABH1 into 5-formylcytidine, which is necessary for reading the AUA codon during protein synthesis.

Methylation and editing of tRNA may require intricate mechanisms and conditions. NSun6, which installs m^5^C72 onto tRNA, recognizes both the sequence and shape of tRNA [92]. Without a folded, full-length tRNA, NSun6 does not methylate m^5^C72. C-to-U deamination of C32 in *Trypanosoma brucei* tRNA^Thr^ also depends on multiple factors [93]. Methylation of C32 to m^3^C by two enzymes, the m^3^C methyltransferase TRM140 and the deaminase ADAT2/3, is a required step in the deamination process. m^3^C must then be deaminated to 3-methyluridine (m^3^U) by the same mechanism, and m^3^U is then demethylated to become U.

The recent discoveries of the first tRNA demethylases, of their effects on translation and differentiation, and of complex mechanisms of tRNA methylation and editing will undoubtedly inspire investigations to elucidate the functions of tRNA modifications and the biological processes to which they respond.

Ribosomal RNA is also marked by abundant modifications; the > 200 modified sites in human rRNAs make up around 2% of rRNA nucleotides. Most modifications on rRNA are Ψ or 2′OMe, although rRNA also contains around ten base modifications [74]. Functions of rRNA modifications are largely unknown, but studies of 2′OMe on rRNA are beginning to provide hints to their functions. The C/D box snoRNAs SNORD14D and SNORD35A, which are necessary to install 2′OMe onto rRNA, are necessary for proper leukemogenesis and are upregulated by leukemia oncogenes [94]. C/D box snoRNA expression in leukemic cells is correlated with protein synthesis and cell size, suggesting a potential role for 2′OMe on rRNA in translation.

The processing and functions of other non-coding RNA species have recently been shown to undergo regulation by m^6^A. Alarcón et al. [95] demonstrated that pri-microRNAs contain m^6^A, which is installed by METTL3 and promotes recognition and processing into mature microRNA by DGCR8. m^6^A is also present on the lncRNA *XIST*, and is necessary for *XIST* to mediate transcriptional silencing on the X chromosome during female mammalian development [96]. Finally, m^6^A is present on human box C/D snoRNA species; it impedes the formation of *trans* Hoogsteen-sugar A–G base pairs, thus affecting snoRNA structure, and also blocks binding by human 15.5-kDa protein [97].

## Concluding remarks and future directions

It is becoming increasingly clear that the epitranscriptome and its modifying enzymes form a complex constellation that holds widely diverse functions. Post-transcriptional RNA modifications allow additional controls of gene expression, serving as powerful mechanisms that eventually affect protein synthesis. In particular, m^6^A provides layers of regulation, offering effects that are dependent on the localization of its writers, readers, and erasers.

To facilitate certain cellular processes, the m^6^A machinery can target multiple substrate mRNAs and non-coding RNAs. As we proposed [38], cellular programs may require a burst of expression of a distinct set of transcripts, followed by expression of a different set of transcripts. m^6^A can mark and cause timely expression and turnover of subsets of transcripts. The cellular and compartmental localizations of the writers, readers, and erasers critically affect their functions. Methylation, together with demethylation of subsets of transcripts in the nucleus, may create a methylation landscape that directs the fate of groups of transcripts as they are processed, exported to the cytoplasm, translated, and degraded. Multiple different readers or their associated proteins may be required to actualize the effects of the methylations fully. Although transcript turnover or decay is an accepted role of mRNA m^6^A methylation, it should be noted that the *Ythdf2* knockout mouse exhibits a less severe phenotype [98] compared to mice lacking *Mettl3* or *Mettl14* (embryonic lethals), demonstrating that the Ythdf2-dependent pathway mediates a subset of the functions of methylated transcripts. There are other crucial regulatory functions of m^6^A RNA methylation that remain to be uncovered.

These observations lead us to perceive that methylation occurs at multiple layers. Methyltransferases set the initial methylation landscape in coordination with the transcription machinery. Demethylases could more efficiently tune the methylation landscape of a subset of methylated transcripts, acting as the second layer of regulation. Indeed, demethylases often target only a subset of genes under certain conditions; for example, depletion of *Alkbh5* does not lead to embryonic lethality but instead causes defects in spermatogenesis [13], and only a portion of *Fto* knockout mice display embryonic lethality. Finally, reader proteins act as effectors in a third layer of regulation, carrying out specific functions upon methylated transcripts.

The field of epitranscriptomics still remains vastly unexplored. Future studies will need to focus on the mechanisms that define which transcripts are methylated. Moreover, as methylations are often unevenly distributed along the RNA transcript, identifying the mechanisms underlying the regional specificity of methylation, as well as which individual sites along transcripts are methylated, remain as major challenges. The methylation selectivity on particular transcripts may need to be coupled with transcription regulation. How this selectivity is determined and the interplay between methylation and transcription require further exploration. Questions regarding the effects of methyltransferases and demethylases on nuclear processing, splicing, and export also remain. Nuclear regulation of RNA methylation could play critical roles impacting biological outcomes. In particular, it will be important to determine how and why a subset of RNAs undergoes demethylation inside the nucleus, as well as the functional consequences of this required demethylation on gene expression. Interactions between the writers, readers, and erasers with other cellular components are also necessary to reveal functional roles, especially those in complex biological processes in vivo.

## Abbreviations

2′OMe: 2′-*O*-methylation
ALKBH5: AlkB homologue 5
AML: Acute myeloid leukemia
ATRA: All-*trans*-retinoic acid
FTO: Fat-mass and obesity-associated protein
GSC: Glioblastoma stem cell
HCC: Hepatocellular carcinoma
HNRNPC: Heterogeneous nuclear ribonucleoprotein C
lncRNA: Long non-coding RNA
m^1^A: *N* ^1^-methyladenosine
m^5^C: 5-methylcytidine
METTL3: Methyltransferase-like 3
mt-tRNA^Met^: Mitochondrial tRNA for methionine
rRNA: Ribosomal RNA
SAM: *S-*adenosyl methionine
*Sxl*: *Sex lethal*
YTHDC1: YTH domain containing 1
YTHDF1: YTH domain family 1
Ψ: Pseudouridine
